# The effects of moderate fatigue on dynamic balance control and attentional demands

**DOI:** 10.1186/1743-0003-3-22

**Published:** 2006-09-28

**Authors:** Martin Simoneau, François Bégin, Normand Teasdale

**Affiliations:** 1 Groupe de Recherche en Analyse du Mouvement et Ergonomie, Faculté de Médecine, Division de kinesiology, Université Laval, Québec, Canada

## Abstract

**Background:**

During daily activities, the active control of balance often is a task per se (for example, when standing in a moving bus). Other constraints like fatigue can add to the complexity of this balance task. In the present experiment, we examined how moderate fatigue induced by fast walking on a treadmill challenged dynamic balance control. We also examined if the attentional demands for performing the balance task varied with fatigue.

**Methods:**

Subjects (n = 10) performed simultaneously a dynamic balance control task and a probe reaction time task (RT) (serving as an indicator of attentional demands) before and after three periods of moderate fatigue (fast walking on a treadmill). For the balance control task, the real-time displacement of the centre of pressure (CP) was provided on a monitor placed in front of the subject, at eye level. Subjects were asked to keep their CP within a target (moving box) moving upward and downward on the monitor. The tracking performance was measured (time spent outside the moving box) and the CP behavior analyzed (mean CP speed and mean frequency of the CP velocity).

**Results:**

Moderate fatigue led to an immediate decrement of the performance on the balance control task; increase of the percentage of time spent outside the box and increase of the mean CP speed. Across the three fatigue periods, subjects improved their tracking performance and reduced their mean CP speed. This was achieved by increasing their frequency of actions; mean frequency of the CP velocity were higher for the fatigue periods than for the no fatigue periods. Fatigue also induced an increase in the attentional demands suggesting that more cognitive resources had to be allocated to the balance task with than without fatigue.

**Conclusion:**

Fatigue induced by fast walking had an initial negative impact on the control of balance. Nonetheless, subjects were able to compensate the effect of the moderate fatigue by increasing the frequency of actions. This adaptation, however, required that a greater proportion of the cognitive resources be allocated to the active control of the balance task.

## Background

Fatigue alters the force capacity of muscles. It is a complex and diverse phenomenon involving neural and muscular mechanisms [1,2]. At the ankle, it decreases the sense of position [3,4] and the control of balance. For example, Lundin et al. [5], have examined how plantar flexor and dorsiflexor fatigue induced through an isokinetic protocol affected the control of balance. They reported a significant increase in medio-lateral (M-L) body sway oscillations amplitude compared with a no fatigued state. Similar observations have been reported by others [6-8].

More global fatigue protocols where fatigue is induced by treadmill walking or skiing, running or cycling also have been used [9-11]. For example, Nardone et al. [9], using a treadmill aerobic fatigue protocol, have reported increases of the sway path of the centre of pressure (CP) and median frequency of the CP velocity after the fatigue protocol. The latter effect suggested the authors that fatigue induces an increased frequency of actions needed to regulate body sway oscillations. Simoneau et al. [11] tested the balance stability of recreational and highly skilled biatheletes in their upright shooting position before and after a metabolic activation similar to that observed in competition. They reported that skilled athletes were less affected by fatigue suggesting that skill could attenuate the specific effect of fatigue on balance control.

Fatigue also alters central processing of proprioception [12,13]. With fatigue, cortico-motor neuronal cells firing rates decrease and motor-evoked potentials increase suggesting inadequate cortical output [see [1], for review]. Besides, central fatigue may induce deterioration of cognitive functions. For example, following mental fatigue, subjects are still able to perform automated tasks but performance in complex tasks deteriorates [14]. Also, when producing submaximal contractions at the elbow, a constant force production can be obtained at the cost of increasing central command intensity. This process is not automatic and Lorist et al. [13] suggested the presence of a mutual interaction between cognitive functions and the central mechanisms driving motor behaviour during fatigue. These authors observed a decline in performance in a dual-task condition (decreased force production and increased probe reaction time) compared to single-task. They suggested that the dual-task condition imposed a 100% workload on the subject's limited attentional demands. Hence, no residual resources were available to compensate for the increasing task demands brought in by the fatigue. Similar interactive processes between cognition and the balance control mechanisms have been suggested [15-17]. For instance, attentional demands are greater for unstable than for stable balance conditions [18-20].

Fatigue does not always lead to task failure. For instance, the term "light work" has been ascribed to work situations in which the task requires low energetic expenditure and in which there are no high peak load to the musculo-skeletal system [21]. In the present study, we wanted to examine how a familiar sub-maximal fatiguing condition, fast walking, modifies balance control. Also because for most daily activities, we not only have to stand in a quasi-static posture but also have to control our CP, which regulates centre of mass (CM) velocity-position [22,23], we have designed a balance task requiring an active control of CP displacements. Based on the work of Lorist et al. [13], we hypothesized the effect of moderate fatigue on balance control could be compensated. This, however, would come at the expense of greater attentional demands.

## Methods

### Results

#### Subjects

Ten healthy young adults (six males and four females, mean age: 22.6 ± 1.7 yr, mean height: 1.72 ± 0.09 cm, and mean weight: 73.6 ± 14.8 kg) participated in the experiment. None of the participants were familiar with the purpose of the experiment. All participants gave written consent according to Laval University ethic committee.

#### Tasks and apparatus

Participants stood on a force platform (AMTI OR6-1), two meters from a 17-in monitor placed at gaze level. The CP was calculated in real-time to provide a direct feedback about the position and displacement through a moving red cross (10 mm × 10 mm). A forward displacement of the subject induced a movement of the cross in the upward direction while a backward displacement created a downward movement of the cross. Left and right movements of the cross corresponded to left and right displacements of the CP. The subject's task consisted of maintaining the red cross within a squared box (20 mm × 20 mm) moving upward and downward for a period of 30 sec.

All subjects were instructed their primary task was to regulate their balance and to keep their CP within the moving box. While performing the postural task, they performed a secondary probe reaction time (RT) task. They responded vocally ("top") as rapidly as possible to an unpredictable auditory stimulus. RT was defined as the temporal interval between the presentation of an auditory stimulus (100 ms, 1.5 kHz) and the onset of the verbal response (detected from the analog signal of a piezoelectric microphone ["Realistic", FM Wireless Microphone System]) mounted on a custom headset. The force platform signals, the auditory stimuli, and the vocal responses were all sampled at 500 Hz (16-bit A/D conversion National Instrument model PCI-6052E).

#### Normalization procedures

Prior to the testing, four different activities, lasting about 45 minutes, took placed: 1) a calibration procedure to determine the amplitude and speed of the moving box, 2) a practice session to learn tracking the moving box, 3) data acquisition of baseline RTs, and 4) a familiarization period with the treadmill also serving to determine the maximal walking speed. These four activities are now described.

**Figure 1:**
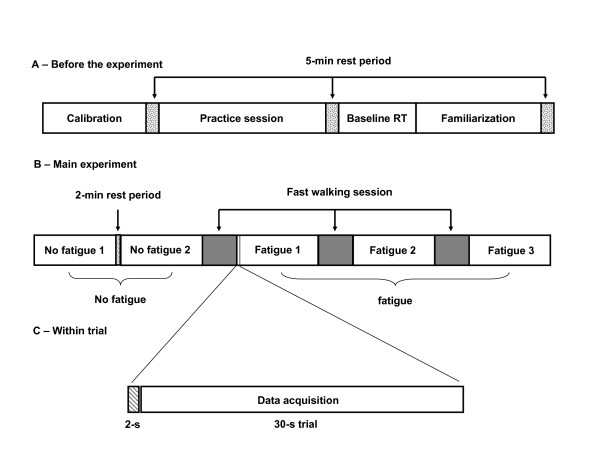
**Schematic representation of the five experimental sessions**. A) Normalization procedure before the experiment. First, the dynamic balance control capabilities of each subject were quantified to determine the amplitude and velocity of the moving box (Calibration). Then, subject rested for 5-min. To avoid possible learning effect during the main experiment, subjects performed 40 trials followed by a 5-min rest period (Practice session). Then, baseline reaction times were collected (Baseline RT). Finally, subjects walked on the treadmill to determine maximum treadmill velocity of each subject (Familiarization). B) Main experiment. The first two blocks are without fatigue (No fatigue 1 and No fatigue 2). There was a 2 minutes rest period between both blocks of no fatigue. The next three blocks composed the fatigue condition (Fatigue 1, Fatigue 2 and Fatigue 3). The gray areas before each of the three block of fatigue correspond to the fast walking periods. After the last block of no fatigue (No fatigue 2), subjects started the first fast walking session (gray area). When they could not keep the pace with the treadmill, they started tracking the moving box for 10 30-s trials. They repeated this procedure twice. C) Within trial. Before each trial, a two seconds period of data acquisition served to align the centre of the moving box with the average centre of pressure calculated during this period. This 2-s period was not included in the centre of pressure data analysis. Each trial lasted 30 seconds.

The calibration procedure allowed setting the amplitude and speed of the moving box (Fig. 1A – Calibration). Subjects were asked to lean forward and backward as far and as fast as possible and to move back to their neutral initial position. When doing so, they were instructed to adopt an inverse pendulum strategy (rotation around the ankle joint and minimize hip and knee movements) and were not allowed to take a step or raise their heels and toes. The amplitude of the trajectory of the moving box along the upward and downward direction was equal to 30% of the maximal backward and forward leaning amplitude, respectively. For all subjects, the mean amplitude of the trajectory was 6.9 cm and it varied from 5.1 to 8.9 cm. The velocity of the box represented 8% of the maximal velocity noted for the backward leaning (that is the minimum of the two maximum velocities performed in the forward-backward leaning). The average speed was 1.86 cm/s. Across subjects, it varied from 0.85 to 3.60 cm/s. For the experimental trials, the box presented on the monitor followed a continuous upward-downward linear trajectory at a constant speed for 30 seconds. By normalizing the target's displacement and speed, we wanted to make sure these parameters would be adjusted to each subject's dynamic balance control capabilities.

During a pilot study (n = 3 subjects), we observed that 40 trials were necessary to attain a stable tracking performance (Time out of the target). For this reason, each subject received 40 practice trials (30 sec each) following the calibration procedure (Fig. 1A – Practice session). Data for these 40 practice trials are not reported herein as their only purpose was to ensure all subjects had attained a stable level of performance prior to the experiment.

A 5-min rest period was then provided and baseline RTs were collected (Fig. 1A – Baseline RT). In the present experiment, subjects were asked to consider the postural task as their primary task. The RT task was the secondary task and any change in RT presumably would reflect changes in the attentional demands necessary to perform the postural task. RT was defined as the temporal interval between the presentation of the auditory stimulus and the onset of the verbal response (detected from a voltage rise in the analog signal of the microphone). For the baseline RTs, subjects were seated comfortably and 12 auditory stimuli were given randomly within a 3-min period. They responded vocally ("top") as rapidly as possible to the auditory stimulus.

Finally, familiarization with the treadmill was provided (Fig. 1A – Familiarization). This also served to determine each subject's maximal walking speed. We started with a treadmill speed of 1.33 m/s and gradually increased it by step of 0.044 m/s until the subject was forced to jog to keep up with the treadmill speed. This also served as a warm-up period. The treadmill speed was then adjusted to the fastest speed observed in the warm-up period. Normal gait speed is around 1.2 m/s. Our subjects walked at speeds nearly twice that fast (from 2.01 m/s to 2.37 m/s, see results section). A 5-min rest period followed these procedures.

#### Experimental procedures

The unfolding of the experimental conditions that followed the normalization procedures is illustrated in Figure 1B. The experiment started with two blocks of 10 30-s tracking trials without fatigue separated by a short rest period (2 min). Before each trial, the participants were asked to keep their arms along their body and stood as still as possible for two seconds. This procedure served to align the centre of the moving box with the average CP calculated for this period. These data were not included in the analysis. Data acquisition (30 sec) followed without any delay. For each trial, 3 or 4 randomly presented auditory stimuli separated by at least 4 sec were given. A total of 35 stimuli were given for each series of 10 trials.

The three walking (fatigue) periods followed. For each fast walking period (gray areas in Fig. 1B), the participants walked on a treadmill (StarTrac, model 3021, Unisen Inc., Tustin, CA, USA) at their maximal speed as long as they could maintain the pace while avoiding running. Subjects were verbally encouraged to maintain their pace. Warnings were given when the participants got beyond a predetermined backward spatial boundary on the treadmill or when they jogged to keep the pace. Each walking period stopped at the second warning. A block of ten balance tracking trials immediately followed. This sequence (fast walking-balance tracking trials) was repeated three times. The duration of the experiment (excluding the normalization procedures) was about 1 hour.

### Data analyses

#### Dynamic balance control

The ability of the participants to control their CP with and without moderate fatigue was determined for each trial by calculating a) the percentage of time spent outside the moving box for both the antero-posterior (A-P) and medio-lateral (M-L) axes, b) the mean speed of the CP, and c) the mean frequency of the CP velocity. Mean speed corresponds to the cumulated distance over the sampling period and it is a good index of the activity required to control balance. Before calculating the mean frequency, we removed the imposed A-P movement of the target from the A-P oscillations. Therefore, the remaining A-P signal consisted of the specific body oscillations of the subjects needed to keep the CP within the moving box. We focused on the CP oscillations along the A-P axis as the moving target moved only along this axis. Then, the derivative of the CP displacement was calculated using a finite difference technique (55-ms weighted window). Power spectra were calculated from smoothed detrended data of the A-P CP velocity using a no overlapping Fast Fourier Transform (FFT) window of 4096 points allowing a resolution of 0.06 Hz. The mean frequency was calculated from the power spectra of each trial to characterize the frequency of actions of the CP velocity.

#### Balance control analyses

Calculated values for each variable were averaged across the ten trials for each block (two no-fatigue and three fatigue blocks). For all dependent variables, a one-way analysis of variance (ANOVA) with repeated measures (i.e., two no-fatigue and three fatigue blocks) was used. Whenever the ANOVA reached a significant level, planned comparisons were used to determine: 1) if the two blocks of no fatigue differed from the three blocks of fatigue, 2) if across fatigue, subjects improved their tracking performance (e.g. decrease of the time spent outside the moving box) and changed their balance control strategy (e.g., increase of their frequency of actions), and 3) if subjects were able to compensate the effect of fatigue (by comparing results for the last block of fatigue with that of the no fatigue blocks. The level of significance was set at P < 0.05. If moderate fatigue decreases dynamic balance control ability, a main effect of block should be observed for all dependant variables (i.e., faster mean speed, longer time spent outside of the moving box and greater frequency of actions for the fatigue compared to the no fatigue block).

#### Reaction time analyses

Balance control is not an automatic task and it requires cognitive resources [15,16,18]. In the present experiment, RT served as an indicator of the cognitive (attentional) demands needed to perform the dynamic balance task. The traditional approach for analyzing RT consists of calculating the mean of a series of trials. There is a growing recognition, however, that a more detailed analysis of the response time distribution provides additional and often critical information that is not available when using more standard statistical summary measures of mean and variance [24-26]. This is particularly the case for human factors research where one is interested not only in the average response but in the slowest response. Often, this slowest response can be associated to a "worst-case" scenario [27]. For instance, the mean RT for responding rapidly to critical information presented on a highway sign is an underestimate of the time necessary to process the information as all trials with slower RT than that of the mean presumably could yield to incorrect motor responses (for example, late braking response or change of trajectory). A similar logic can be applied to the present experiment where a) the fastest RTs could allow to determine if the capability of responding as rapidly as possible is maintained with fatigue, and b) the slowest RTs could provide an indication of the "worst-case scenario" where the attentional demands necessary to regulate the body sway oscillations have exceeded the normal operating range. For this reason, we analyzed the 10^th ^and 90^th ^percentile RT for each block of data. These values correspond more or less to the fastest and slowest RT observed for a block of data. RT data were submitted to a two-way ANOVA (Percentile × Block) with repeated measures on the factor Block. Slower RTs with fatigue would indicate a greater reliance upon the cognitive process necessary for body sway oscillations.

For one subject, RTs for trials after the second treadmill period, failed to be recorded because the piezoelectric microphone was turned off inadvertently. Hence, for this subject only, RTs data for the first fatigue block were missing. Further, two responses (4.068 s and 3.428 s) from another subject were removed because the subject reported after the trial that he simply had forgotten answering to the auditory stimuli.

## Results

### Walking duration across the three fatigue periods

Subjects walked at a speed varying from 2.01 m/s to 2.37 m/s. The walking duration decreased from the first to the third period (on average, 10:27, 07:28, 06:06 minutes for the first, second and third walking period, respectively). A one-way ANOVA (three treadmill walking periods) showed that this decrease was statistically significant (F_2,18 _= 5.48, P < 0.05) suggesting our walking protocol induced some fatigue.

### Dynamic balance control performance

**Figure 2:**
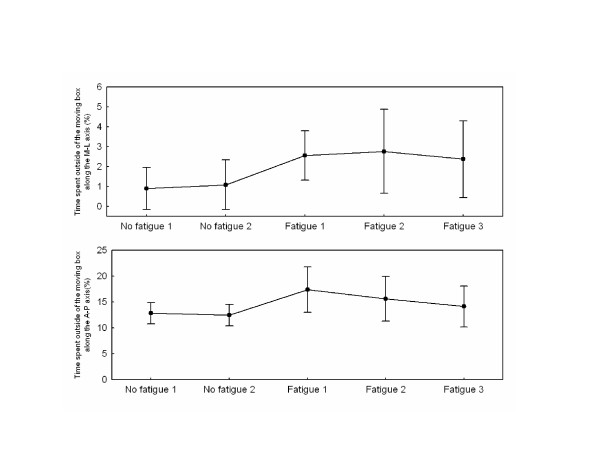
**Participant's performance**. Upper panel and lower panels show the percentage of time the CP spent outside of the moving box along the M-L and the A-P axis for all blocks. The error bars represent 0.95 confidence intervals.

Results for the time spent outside of the moving box for both M-L and A-P axes are illustrated in Fig. 2 (upper and lower panels, respectively). The ANOVAs showed significant effects of Block (F_4,36 _= 4.72, P < 0.01 and F_4,36 _= 6.15, P < 0.001 for A-P and M-L, respectively). Planned comparisons between the no fatigue and fatigue conditions showed a significant effect of fatigue for both directions (F_1,9 _= 5.39, P < 0.05 and F_1,9 _= 33.03, P < 0.001 for A-P and M-L direction, respectively). Without fatigue, for the A-P direction, subjects spent on average 12.8% and 12.4% of the 30-s outside the moving box. With fatigue, the percentages were 17.4%, 15.6% and 14.1%, respectively. A comparison of the three values with fatigue showed the tracking performance improved gradually across the three blocks with fatigue (F_1,9 _= 13.91, P < 0.05). Also, the performance for the last block with fatigue was not different from that observed without fatigue (F_1,9 _= 1.40, P > 0.05) suggesting that, across the blocks of fatigue, subjects were able to improve their tracking performance. For the M-L data, there was no improvement across the blocks of fatigue (F_1,9 _= 0.07, P > 0.05). The mean scores for the M-L direction, however, were considerably smaller than scores for the A-P direction (0.9%, 1.1%, 2.6%, 2.8% and 2.4% for the five blocks, respectively).

**Figure 3:**
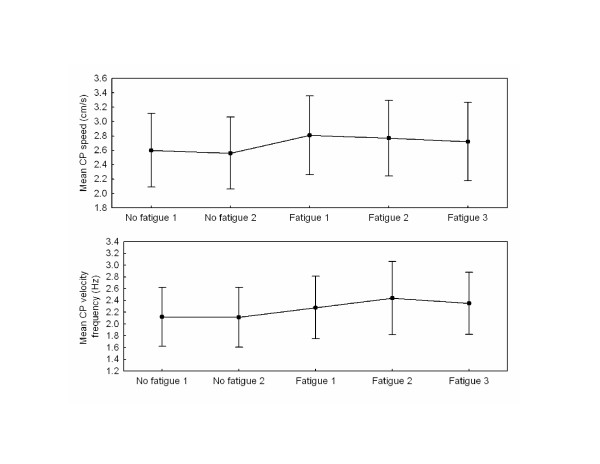
**Mean CP speed and Frequency of action**. Upper panel presents mean speed of the center of pressure for all blocks. Lower panel illustrates mean frequency of the centre of pressure velocity for all blocks. The error bars on both panels represent 0.95 confidence intervals.

Results for mean speed are presented in Fig. 3 – upper panel. The main effect of Block was significant (F_4,36 _= 9.05, P < 0.001) and a planned comparison between the no fatigue and fatigue blocks showed a significant effect of fatigue (F_1,9 _= 18.49, P < 0.01). Speed for the last block with fatigue was greater from that observed for the last block without fatigue (F_1,9 _= 19.55, P < 0.01).

### Frequency of actions of the CP velocity

The ANOVAs for the mean frequency (Fig. 3 – lower panel) revealed an effect of Block (F_4,36 _= 7.93, P < 0.001). The planned comparison between the no fatigue and fatigue conditions showed a significant effect of fatigue (F_1,9 _= 16.41, P < 0.01). The frequency of actions increased between the first and the second block of fatigue (F_1,9 _= 6.06, P < 0.05). The difference between the last block of fatigue and the last block without fatigue also was significant (F_1,9 _= 7.22, P < 0.05). This suggests that participants increased their frequency of actions to compensate the effect of fatigue. This change in behavior, to some extent, helped the subjects increased their tracking performance.

### Cognitive processing

**Figure 4:**
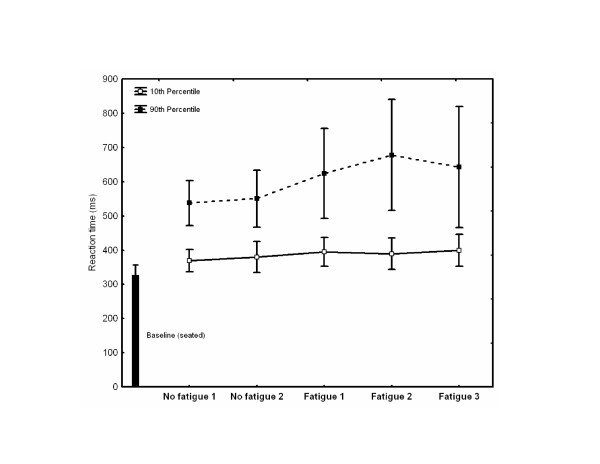
**Attentional demands**. Mean reaction time for the 10th (□) and 90th (■) percentiles. The error bars represent 0.95 confidence intervals.

Results for the 10^th ^and 90^th ^percentile RTs for each block are presented in Fig. 4. The ANOVA showed a significant interaction of Percentile by Block (F_4,36 _= 3.14, P < 0.05). RTs for the 10^th ^percentile did not vary across the five blocks of trials (F_4,36 _= 2.33, P > 0.05), suggesting subjects were able to produce rapid responses and that moderate fatigue did not alter this capacity. The 10^th ^percentile RT was, on average, 386 ms. Baseline (seated) RT was, on average, 327 ms. With moderate fatigue, however, RT for the 90^th ^percentile increased significantly from the no-fatigue to the fatigue condition (F_4,36 _= 3.60, P < 0.05). This suggests that moderate fatigue yielded an increase in the attentional demands necessary for regulating body sway oscillations. A comparison of the three RT values with fatigue showed that the cognitive demands did not change across the three blocks with fatigue (F_1,8 _= 1.40, P > 0.05 and F_1,8 _= 0.01, P > 0.05 for the comparisons between the first and second and second and third blocks, respectively).

## Discussion

The aim of this study was to examine the effect of moderate fatigue induced by fast walking periods on the dynamic control of balance. A task requiring an active control of the CP was developed. A first observation is that moderate fatigue had an initial detrimental effect on the control of balance; it yielded a significant increase of the time spent outside of the moving box along the A-P axis. This decline of performance agrees with previous studies concerning the effect of strenuous fatigue on the control of upright standing posture [6-9]. An interesting observation from the present results concerns the gradual decrease of the time spent outside of the target (moving box) through the three blocks of moderate fatigue. The improvement of the tracking performance along the A-P direction suggests that subjects adapted their balance control mechanisms to cope with moderate fatigue. This result cannot be attributed to learning the balance task as subjects were familiarized with it before starting the experiment and the performance of all subjects was stable before the fast walking sessions started. In fact, all subjects received a total of 60 30-sec trials (40 familiarization and 20 experimental trials) before starting with the fast walking protocol. Results from the frequency analyses of the CP velocity also clearly suggest the participants increased their frequency of actions (i.e., increase of the mean frequency of the CP velocity) to better track the moving box. The mean frequency increased after the first block of fatigue and this increased frequency was associated with an improvement of the tracking performance. Similar control strategies (increase in postural frequencies > 0.5 Hz) have been proposed after a global fatigue protocol induced by treadmill running [9]. Despite the fact that, in the above experiment, subjects did not have to track a moving target with their CP (and CM), there has been suggestions that higher frequencies reveal the neuromuscular activity used to counteract fatigue effects [9,28] and maintain the CP within stable boundaries.

The initial deterioration and subsequent improvement of the performance for the dynamic balance task (tracking performance) could be attributed in part to the detection/action capability of the central nervous system. With fatigue, muscle spindles tend to decrease their firing rate [29] and the cortico-motor neuronal cells firing decreases and become more irregular [30,31]. Furthermore, fatigue induces greater variability or noise in the afferent signal [3]. Altogether, these initial changes could result to a poorer detection of the CP position. Across trials of moderate fatigue, however, Ia afferent fibers could become more sensitive to muscle fibers length changes. Studies on animal muscle spindles showed that Ia afferent fibers could still discharge at higher rates to local stretch on their receptors even with the decline of their firing rates caused by muscular fatigue [32,33]. With fatigue, more extrafusal fibers are loaded. Muscle spindles that are sensitive to the discharge of neighboring motor units might consequently increase their firing rates [1,34]. Hence, muscle spindles could be able to drive effectively changes of muscle length under moderate physical activity and the dynamic balance control system could better identify noise from *real *proprioceptive signal. Besides, following the first fatigue period the transformation of noisy sensory inputs into balance control commands could be inappropriate. Across fatigue periods, however, an improvement of the sensory detection capabilities may help the brain selecting balance control commands leading to better tracking performance. From a practical viewpoint, the improvement of the dynamic balance control task through moderate fatigue suggests that balance training performed with fatigue could be beneficial [35]. Previous results comparing the postural stability of recreational and highly skilled athletes after a strenuous effort also support this suggestion [11]. In that experiment, skilled athletes were less affected by fatigue than recreational biathletes.

In the present experiment, RTs served as an indicator of cognitive processing for controlling the CP [15,16,18]. This was proposed because unstable balance conditions have been shown to require more attentional demands than stable balance conditions [18-20]. Even though an improvement of the dynamic balance control task was observed through blocks of moderate fatigue, the slower RTs (90^th ^percentile RT) observed across all three blocks of fatigue suggest that greater cognitive processing was needed to dynamically control the CP. The allocation of cognitive processes certainly is not static. Indeed, dynamic allocation of the resources to the postural task and task sharing strategies have been proposed [15,36-38]. The analyses of the 10^th ^percentile and 90^th ^percentile RT data support this suggestion. On one hand, values for 10^th ^percentile showed that the capacity to respond rapidly was not affected by moderate fatigue since RTs were not different across all blocks (but did require cognitive resources since baseline RT was faster than values for the 10^th ^percentile RT). On the other hand, values for the 90^th ^percentile showed large increases for the moderate fatigue conditions suggesting that we were able to capture transient events where subjects allocated a greater portion of their cognitive resources to the dynamic balance control task. A more direct examination of this process would require to analyze RTs as a function of whether the CP was within or outside the moving box (or as Teasdale et al. [18] did when the CP moved towards or away from a stable mean CP position). Unfortunately, the present experiment was not designed to examine this particular issue and the small number of trials available for each subject did not allow us to conduct this specific analysis. As Lorist et al. [13] suggested for a force production task at the elbow, it may well be that slower RTs observed with fatigue reflects the attentional demands necessary to increase the central command intensity or to increase the firing rates of the motor units [1]. The mutual interaction between the allocation of cognitive processing and balance control in a fatigued state is clearly a topic that will require future research.

## Conclusion

Overall, the present study shows that moderate fatigue induced by fast walking on a treadmill has a detrimental initial impact on the control of CP. The dynamic balance control system, though, is able to compensate the early acute effects of fatigue by increasing the frequency of actions of the CP velocity and allocating a greater portion of the cognitive resources to the balance control task. This strategy allowed subjects to increase their balance control performance by decreasing the time spent outside of the moving box across blocks of fatigue. Altogether, this suggests that subjects can learn to manage detrimental effects of sub-maximal fatiguing conditions. This observation could contribute to the development of occupational interventions aimed at mitigating the effect of fatigue on balance control.

## Competing interests

The author(s) declare that they have no competing interests.

## Authors' contributions

FB recruited subjects, managed data acquisition and participated to data analysis and drafting of the manuscript. MS and NT conceived the study, evaluated the data, performed data analyses and wrote the manuscript.
